# Supplementary material for: Asymptotically local synchronization in interdependent networks with unidirectional interlinks
Source: PLoS One. 2022 May 5;17(5):e0267909. doi: 10.1371/journal.pone.0267909 (PMC9070916; doi:10.1371/journal.pone.0267909)
Supplement: S1 Appendix — (DOCX) [file pone.0267909.s001.docx]

**APPENDIX 1**

All equations in the manuscript are shown as follows. The proof of Theorem 1 is also shown in details.

$$\begin{aligned} \dot{x}_{i}=f\left( x_{i} \right)+\alpha\sum_{j=1}^{N} a_{ij}^{x}H\left( x_{j} \right) i=1,2,\ldots,N\#\left( 1 \right) \end{aligned}$$

$$\begin{aligned} \dot{y}_{i}=g\left( y_{i} \right)+\beta\sum_{j=1}^{N} a_{ij}^{y}K\left( y_{j} \right)+\gamma\sum_{j=1}^{N} c_{ij}\left( H\left( x_{j} \right)-K\left( y_{i} \right) \right) i=1,2,\ldots,N\#\left( 2 \right) \end{aligned}$$

$$\begin{aligned} \dot{x}_{i}=f\left( x_{i} \right)+\alpha\sum_{j=1}^{N} a_{ij}^{x}H\left( x_{j} \right)+u_{i}^{x} i=1,2,\ldots,N\#\left( 3 \right) \end{aligned}$$

$$\begin{aligned} \dot{y}_{i}=g\left( y_{i} \right)+\beta\sum_{j=1}^{N} a_{ij}^{y}K\left( y_{j} \right)+\gamma\sum_{j=1}^{N} c_{ij}\left( H\left( x_{j} \right)-K\left( y_{i} \right) \right)+u_{i}^{y} i=1,2,\ldots,N\#\left( 4 \right) \end{aligned}$$

$$\begin{aligned} \dot{s}^{x}\left( t \right)=f\left( s^{x}\left( t \right) \right)\#\left( 5 \right) \end{aligned}$$

$$\begin{aligned} \dot{s}^{y}\left( t \right)=g\left( s^{y}\left( t \right) \right)\#\left( 6 \right) \end{aligned}$$

$$\begin{aligned} \lim_{t\to\infty} \left\| x_{i}-s^{x}\left( t \right) \right\|=0, i=1,2,\ldots,N\#\left( 7 \right) \end{aligned}$$

$$\begin{aligned} \lim_{t\to\infty} \left\| y_{i}-s^{y}\left( t \right) \right\|=0, i=1,2,\ldots,N\#\left( 8 \right) \end{aligned}$$

$$\begin{aligned} e_{i}^{x}=x_{i}-s^{x}\left( t \right)\#\left( 9 \right) \end{aligned}$$

$$\begin{aligned} e_{i}^{y}=y_{i}-s^{y}\left( t \right)\#\left( 10 \right) \end{aligned}$$

$$\dot{e}_{i}^{x}=\dot{x}_{i}-\dot{s}^{x}\left( t \right)$$

$$=f\left( x_{i} \right)-f\left( s^{x}\left( t \right) \right)+\alpha\sum_{j=1}^{N} a_{ij}^{x}H\left( x_{j} \right)+u_{i}^{x} (11)$$

$$\dot{e}_{i}^{y}=\dot{y}_{i}-\dot{s}^{y}\left( t \right)$$

$$=g\left( y_{i} \right)-g\left( s^{y}\left( t \right) \right)+\beta\sum_{j=1}^{N} a_{ij}^{y}K\left( y_{j} \right)+\gamma\sum_{j=1}^{N} c_{ij}\left( H\left( x_{j} \right)-K\left( y_{i} \right) \right)+u_{i}^{y} (12)$$

$$\begin{aligned} u_{i}^{x}=-\alpha\sum_{j=1}^{N} a_{ij}^{x}H\left( s^{x}\left( t \right) \right)+d_{i}^{x}e_{i}^{x}\#\left( 13 \right) \end{aligned}$$

$$\begin{aligned} u_{i}^{y}=-\beta\sum_{j=1}^{N} a_{ij}^{y}K\left( s^{y}\left( t \right) \right)-\gamma\sum_{j=1}^{N} c_{ij}H\left( s^{x}\left( t \right) \right)+\gamma\sum_{j=1}^{N} c_{ij}K\left( s^{y}\left( t \right) \right)+d_{i}^{y}e_{i}^{y}\#\left( 14 \right) \end{aligned}$$

$$\begin{aligned} \dot{d}_{i}^{x}=-k_{i}^{x}e_{i}^{xT}e_{i}^{x}\#\left( 15 \right) \end{aligned}$$

$$\begin{aligned} \dot{d}_{i}^{y}=-k_{i}^{y}e_{i}^{yT}e_{i}^{y}\#\left( 16 \right) \end{aligned}$$

**Proof.** Introduce (13), (14) into error system (11) and (12) respectively and use linearization method.

$$\dot{e}_{i}^{x}=f\left( x_{i} \right)-f\left( s^{x}\left( t \right) \right)+\alpha\sum_{j=1}^{N} a_{ij}^{x}H\left( x_{j} \right)-\alpha\sum_{j=1}^{N} a_{ij}^{x}H\left( s^{x}\left( t \right) \right)+d_{i}^{x}e_{i}^{x}$$

$$=F\left( t \right)e_{i}^{x}+\alpha\sum_{j=1}^{N} a_{ij}^{x}B\left( t \right)e_{j}^{x}+d_{i}^{x}e_{i}^{x} (17)$$

$$\dot{e}_{i}^{y}=g\left( y_{i} \right)-g\left( s^{y}\left( t \right) \right)+\beta\sum_{j=1}^{N} a_{ij}^{y}K\left( y_{j} \right)+\gamma\sum_{j=1}^{N} c_{ij}\left( H\left( x_{j} \right)-K\left( y_{i} \right) \right)-\beta\sum_{j=1}^{N} a_{ij}^{y}K\left( s^{y}\left( t \right) \right)-\gamma\sum_{j=1}^{N} c_{ij}H\left( s^{x}\left( t \right) \right)+\gamma\sum_{j=1}^{N} c_{ij}K\left( s^{y}\left( t \right) \right)+d_{i}^{y}e_{i}^{y}$$

$$=G\left( t \right)e_{i}^{y}+\beta\sum_{j=1}^{N} a_{ij}^{y}D\left( t \right)e_{j}^{y}+\gamma\sum_{j=1}^{N} c_{ij}B\left( t \right)e_{j}^{x}-\gamma\sum_{j=1}^{N} c_{ij}D\left( t \right)e_{i}^{y}+d_{i}^{y}e_{i}^{y} (18)$$

Let $\lambda_{max}^{F}$ be the maximum eigenvalue of matrix $(F^{T}+F)$, $\lambda_{max}^{G}$ be the maximum eigenvalue of matrix $(G^{T}+G)$, $\lambda_{max}^{B}$ be the maximum eigenvalue of matrix $BB^{T}$, $\lambda_{max}^{D}$ be the maximum eigenvalue of matrix $DD^{T}$. Let $\tilde{a}_{1}^{x}=\max_{1\leq i\leq N} \left| a_{ij}^{x} \right|, \tilde{a}_{2}^{x}=\max_{1\leq i\leq N} \left| a_{ji}^{x} \right|, \tilde{a}_{1}^{y}=\max_{1\leq i\leq N} \left| a_{ij}^{y} \right|, \tilde{a}_{2}^{y}=\max_{1\leq i\leq N} \left| a_{ji}^{y} \right|, \tilde{c}_{1}=\max_{1\leq i\leq N} \left| c_{ij} \right|, \tilde{c}_{2}=\max_{1\leq i\leq N} \left| c_{ji} \right|.$

Choose the candidate Lyapunov function

$$\begin{aligned} V\left( t \right)=\sum_{i=1}^{N} e_{i}^{xT}e_{i}^{x}+\sum_{i=1}^{N} e_{i}^{yT}e_{i}^{y}+\sum_{i=1}^{N} \frac{\left( d_{i}^{x}+d_{*}^{x} \right)^{2}}{k_{i}^{x}}+\sum_{i=1}^{N} \frac{\left( d_{i}^{y}+d_{*}^{y} \right)^{2}}{k_{i}^{y}}\#\left( 19 \right) \end{aligned}$$

where $d_{*}^{x},d_{*}^{y}$ are constant, and satisfy $d_{*}^{x}>\left( \lambda_{max}^{F}+\alpha N\tilde{a}_{1}^{x}\lambda_{max}^{B}+\alpha N\tilde{a}_{2}^{x}+\gamma N\tilde{c}_{2} \right)/2$, $d_{*}^{y}>\left( \lambda_{max}^{G}+\beta N\tilde{a}_{1}^{y}\lambda_{max}^{D}+\gamma N\tilde{c}\lambda_{max}^{B}+\beta N\tilde{a}_{2}^{y} \right)/2$.

Then, with equation (15) and (16), the derivative of $V\left( t \right)$ along error system (17) and (18) is obtained as

$$\dot{V}\left( t \right)=\sum_{i=1}^{N} {(\dot{e}}_{i}^{xT}e_{i}^{x}+e_{i}^{xT}\dot{e}_{i}^{x})+\sum_{i=1}^{N} \left( \dot{e}_{i}^{yT}e_{i}^{y}+e_{i}^{yT}\dot{e}_{i}^{y} \right)+2\sum_{i=1}^{N} \frac{d_{i}^{x}+d_{*}^{x}}{k_{i}^{x}}\dot{d}_{i}^{x}+2\sum_{i=1}^{N} \frac{d_{i}^{y}+d_{*}^{y}}{k_{i}^{y}}\dot{d}_{i}^{y}$$

$$=\sum_{i=1}^{N} \left( \left( F\left( t \right)e_{i}^{x}+\alpha\sum_{j=1}^{N} a_{ij}^{x}B\left( t \right)e_{j}^{x}+d_{i}^{x}e_{i}^{x} \right)^{T}e_{i}^{x}+e_{i}^{xT}\left( F\left( t \right)e_{i}^{x}+\alpha\sum_{j=1}^{N} a_{ij}^{x}B\left( t \right)e_{j}^{x}+d_{i}^{x}e_{i}^{x} \right)+\left( G\left( t \right)e_{i}^{y}+\beta\sum_{j=1}^{N} b_{ij}D\left( t \right)e_{j}^{y}+\gamma\sum_{j=1}^{N} c_{ij}B\left( t \right)e_{j}^{x}-\gamma\sum_{j=1}^{N} c_{ij}D\left( t \right)e_{i}^{y}+d_{i}^{y}e_{i}^{y} \right)^{T}e_{i}^{y}+e_{i}^{yT}\left( G\left( t \right)e_{i}^{y}+\beta\sum_{j=1}^{N} a_{ij}^{y}D\left( t \right)e_{j}^{y}+\gamma\sum_{j=1}^{N} c_{ij}B\left( t \right)e_{j}^{x}-\gamma\sum_{j=1}^{N} c_{ij}D\left( t \right)e_{i}^{y}+d_{i}^{y}e_{i}^{y} \right) \right)-2\sum_{i=1}^{N} \left( d_{i}^{x}+d_{*}^{x} \right)e_{i}^{xT}e_{i}^{x}-2\sum_{i=1}^{N} \left( d_{i}^{y}+d_{*}^{y} \right)e_{i}^{yT}e_{i}^{y}$$

$$=\sum_{i=1}^{N} \left( e_{i}^{xT}F^{T}\left( t \right)e_{i}^{x}+\alpha\sum_{j=1}^{N} a_{ij}^{x}e_{j}^{xT}B^{T}\left( t \right)e_{i}^{x}+e_{i}^{xT}F\left( t \right)e_{i}^{x}+\alpha\sum_{j=1}^{N} a_{ij}^{x}e_{i}^{xT}B\left( t \right)e_{j}^{x}+e_{i}^{yT}G^{T}\left( t \right)e_{i}^{y}+\beta\sum_{j=1}^{N} a_{ij}^{y}e_{j}^{yT}D^{T}\left( t \right)e_{i}^{y}+\gamma\sum_{j=1}^{N} c_{ij}e_{j}^{xT}B^{T}\left( t \right)e_{i}^{y}-\gamma\sum_{j=1}^{N} c_{ij}e_{i}^{yT}D^{T}\left( t \right)e_{i}^{y}+e_{i}^{yT}G\left( t \right)e_{i}^{y}+\beta\sum_{j=1}^{N} a_{ij}^{y}e_{i}^{yT}D\left( t \right)e_{j}^{y}+\gamma\sum_{j=1}^{N} c_{ij}e_{i}^{yT}B\left( t \right)e_{j}^{x}-\gamma\sum_{j=1}^{N} c_{ij}e_{i}^{yT}D\left( t \right)e_{i}^{y}-2d_{*}^{x}e_{i}^{xT}e_{i}^{x}-2d_{*}^{y}e_{i}^{yT}e_{i}^{y} \right) (20)$$

According to lemma 1, we can get the results as follows:

$$\alpha\sum_{j=1}^{N} a_{ij}^{x}e_{j}^{xT}B^{T}\left( t \right)e_{i}^{x}+\alpha\sum_{j=1}^{N} a_{ij}^{x}e_{i}^{xT}B\left( t \right)e_{j}^{x}\leq\alpha\sum_{j=1}^{N} \left| a_{ij}^{x} \right|\left( e_{j}^{xT}e_{j}^{x}+e_{i}^{xT}B\left( t \right)B^{T}\left( t \right)e_{i}^{x} \right)=\alpha\sum_{j=1}^{N} \left| a_{ji}^{x} \right|e_{i}^{xT}e_{i}^{x}+\alpha\sum_{j=1}^{N} \left| a_{ij}^{x} \right|e_{i}^{xT}B\left( t \right)B^{T}\left( t \right)e_{i}^{x} (21)$$

$$\beta\sum_{j=1}^{N} a_{ij}^{y}e_{j}^{yT}D^{T}\left( t \right)e_{i}^{y}+\beta\sum_{j=1}^{N} a_{ij}^{y}e_{i}^{yT}D\left( t \right)e_{j}^{y}\leq\beta\sum_{j=1}^{N} \left| a_{ij}^{y} \right|\left( e_{j}^{yT}e_{j}^{y}+e_{i}^{yT}D\left( t \right)D^{T}\left( t \right)e_{i}^{y} \right)=\beta\sum_{j=1}^{N} \left| a_{ji}^{y} \right|e_{i}^{yT}e_{i}^{y}+\beta\sum_{j=1}^{N} \left| a_{ij}^{y} \right|e_{i}^{yT}D\left( t \right)D^{T}\left( t \right)e_{i}^{y} (22)$$

$$\gamma\sum_{j=1}^{N} c_{ij}e_{j}^{xT}B^{T}\left( t \right)e_{i}^{y}+\gamma\sum_{j=1}^{N} c_{ij}e_{i}^{yT}B\left( t \right)e_{j}^{x}\leq\gamma\sum_{j=1}^{N} \left| c_{ij} \right|\left( e_{j}^{xT}e_{j}^{x}+e_{i}^{yT}B\left( t \right)B^{T}\left( t \right)e_{i}^{y} \right)=\gamma\sum_{j=1}^{N} \left| c_{ji} \right|e_{i}^{xT}e_{i}^{x}+\gamma\sum_{j=1}^{N} \left| c_{ij} \right|e_{i}^{yT}B\left( t \right)B^{T}\left( t \right)e_{i}^{y} (23)$$

Introduce (21)-(23) into (20), and with assumptions 1-2 $\dot{V}\left( t \right)$ can be rewritten as

$$\dot{V}\left( t \right)\leq\sum_{i=1}^{N} \left( e_{i}^{xT}F^{T}\left( t \right)e_{i}^{x}+\alpha\sum_{j=1}^{N} \left| a_{ji}^{x} \right|e_{i}^{xT}e_{i}^{x}+e_{i}^{xT}F\left( t \right)e_{i}^{x}+\alpha\sum_{j=1}^{N} \left| a_{ij}^{x} \right|e_{i}^{xT}B\left( t \right)B^{T}\left( t \right)e_{i}^{x}+e_{i}^{yT}G^{T}\left( t \right)e_{i}^{y}+\beta\sum_{j=1}^{N} \left| a_{ji}^{y} \right|e_{i}^{yT}e_{i}^{y}+\gamma\sum_{j=1}^{N} \left| c_{ji} \right|e_{i}^{xT}e_{i}^{x}-\gamma\sum_{j=1}^{N} c_{ij}e_{i}^{yT}D^{T}\left( t \right)e_{i}^{y}+e_{i}^{yT}G\left( t \right)e_{i}^{y}+\beta\sum_{j=1}^{N} \left| a_{ij}^{y} \right|e_{i}^{yT}D\left( t \right)D^{T}\left( t \right)e_{i}^{y}+\gamma\sum_{j=1}^{N} \left| c_{ij} \right|e_{i}^{yT}B\left( t \right)B^{T}\left( t \right)e_{i}^{y}-\gamma\sum_{j=1}^{N} c_{ij}e_{i}^{yT}D\left( t \right)e_{i}^{y}-2d_{*}^{x}e_{i}^{xT}e_{i}^{x}-2d_{*}^{y}e_{i}^{yT}e_{i}^{y} \right)$$

$$=\sum_{i=1}^{N} \left( e_{i}^{xT}\left( F^{T}\left( t \right)+F\left( t \right)+\alpha\sum_{j=1}^{N} \left| a_{ij}^{x} \right|B\left( t \right)B^{T}\left( t \right)+\alpha\sum_{j=1}^{N} \left| a_{ji}^{x} \right|+\gamma\sum_{j=1}^{N} \left| c_{ji} \right|-2d_{*}^{x} \right)e_{i}^{x}+e_{i}^{yT}\left( G^{T}\left( t \right)+G\left( t \right)+\beta\sum_{j=1}^{N} \left| a_{ij}^{y} \right|D\left( t \right)D^{T}\left( t \right)+\gamma\sum_{j=1}^{N} \left| c_{ij} \right|B\left( t \right)B^{T}\left( t \right)+\beta\sum_{j=1}^{N} \left| a_{ji}^{y} \right|-\gamma\sum_{j=1}^{N} c_{ij}\left( D^{T}\left( t \right)+D\left( t \right) \right)-2d_{*}^{y} \right)e_{i}^{y} \right)$$

$$\leq\sum_{i=1}^{N} e_{i}^{xT}\left( F^{T}+F+\alpha\sum_{j=1}^{N} \left| a_{ij}^{x} \right|BB^{T}+\alpha\sum_{j=1}^{N} \left| a_{ji}^{x} \right|+\gamma\sum_{j=1}^{N} \left| c_{ji} \right|-2d_{*}^{x} \right)e_{i}^{x}+\sum_{i=1}^{N} e_{i}^{yT}\left( G^{T}+G+\beta\sum_{j=1}^{N} \left| a_{ij}^{y} \right|DD^{T}+\gamma\sum_{j=1}^{N} \left| c_{ij} \right|BB^{T}+\beta\sum_{j=1}^{N} \left| a_{ji}^{y} \right|-\gamma\sum_{j=1}^{N} c_{ij}\left( D^{T}+D \right)-2d_{*}^{y} \right)e_{i}^{y}$$

$$<\left( \lambda_{max}^{F}+\alpha N\tilde{a}_{1}^{x}\lambda_{max}^{B}+\alpha N\tilde{a}_{2}^{x}+\gamma N\tilde{c}_{2}-2d_{*}^{x} \right)\sum_{i=1}^{N} e_{i}^{xT}e_{i}^{x}+\left( \lambda_{max}^{G}+\beta N\tilde{a}_{1}^{y}\lambda_{max}^{D}+\gamma N\tilde{c}\lambda_{max}^{B}+\beta N\tilde{a}_{2}^{y}-2d_{*}^{y} \right)\sum_{i=1}^{N} e_{i}^{yT}e_{i}^{y} (24)$$

Note that $d_{*}^{x}>\left( \lambda_{max}^{F}+\alpha N\tilde{a}_{1}^{x}\lambda_{max}^{B}+\alpha N\tilde{a}_{2}^{x}+\gamma N\tilde{c}_{2} \right)/2$and $d_{*}^{y}>\left( \lambda_{max}^{G}+\beta N\tilde{a}_{1}^{y}\lambda_{max}^{D}+\gamma N\tilde{c}\lambda_{max}^{B}+\beta N\tilde{a}_{2}^{y} \right)/2$, so $\dot{V}\left( t \right)<0$.

The proof is completed.
